# Supplementary material for: Resolving the full spectrum of human genome variation using Linked-Reads
Source: Genome Res. 2019 Apr;29(4):635–45. doi: 10.1101/gr.234443.118 (PMC6442396; doi:10.1101/gr.234443.118)
Supplement: Supplemental Material [file supp_gr.234443.118_Supplemental_longranger-2.2.zip › longranger-2.2.tar.bz2!/lr2.2-public/lib/go/src/code.google.com/vitess/docs/terms/index.html]

Vitess / Terms & Conditions


Toggle navigation

- Overview
- Guides
- Reference
- Blog
- GitHub

- #### Overview

  - What is Vitess
  - Scaling MySQL with Vitess
  - Key Concepts
- #### Getting Started

  - Run Vitess on Kubernetes
    - Custom Docker Build
  - Run Vitess Locally
- #### User Guide

  - Introduction- Client Libraries- Backing Up Data- Reparenting
        - Schema Management
        - VSchema Guide
        - Schema Swap (Tutorial)
        - Sharding
          - Horizontal Sharding (Tutorial, automated)
          - Horizontal Sharding (Tutorial, manual)
          - Sharding in Kubernetes (Tutorial, automated)
          - Sharding in Kubernetes (Tutorial, manual)
        - Vitess Sequences
        - MySQL Server Protocol
        - Vitess and Replication
        - Update Stream
        - Row Based Replication
        - Topology Service
        - Transport Security Model
        - Launching
          - Scalability Philosophy
          - Production Planning
          - Server Configuration
          - 2PC Guide
          - Troubleshooting
        - Upgrading
- #### Reference Guides

  - Vitess API- vtctl Commands
- #### Other Resources

  - Presentations- Blog- Roadmap
- #### Contributing

  - Contributing to Vitess- GitHub Workflow- Code Reviews
- GitHub

# Terms & Conditions

# Terms & Conditions

All content provided is for informational purposes only. The articles and posts on this website are my own and don't necessarily represent the positions, strategies, or opinions of my employer or its subsidiaries. I make no representations as to the accuracy or completeness of any information found here or by following any links. I will not be liable for any errors or omissions in this information nor for the availability of this information. I will not be liable for any losses, injuries, or damages from the display or use of this information.

## Privacy Policy

The privacy of my visitors is extremely important. This Privacy Policy outlines the types of personal information that is received and collected and how it is used.

First and foremost, I will never share your email address or any other personal information to anyone without your direct consent.

### Log Files

Like many other websites, this site uses log files to help learn about when, from where, and how often traffic flows to this site. The information in these log files include:

- Internet Protocol addresses (IP)
- Types of browser
- Internet Service Provider (ISP)
- Date and time stamp
- Referring and exit pages
- Number of clicks

All of this information is not linked to anything that is personally identifiable.

### Cookies and Web Beacons

When you visit this site "convenience" cookies are stored on your computer when you submit a comment to help you log in faster to Disqus the next time you leave a comment.

Third-party advertisers may also place and read cookies on your browser and/or use web beacons to collect information. This site has no access or control over these cookies. You should review the respective privacy policies on any and all third-party ad servers for more information regarding their practices and how to opt-out.

If you wish to disable cookies, you may do so through your web browser options. Instructions for doing so can be found on the specific web browsers' websites.

## Disclosure Policy

I make money on this website through affiliate programs. If you click an affiliate link or ad banner and buy the product, you help support this website because I'll get a percentage of that sale.

Currently I'm an affiliate for Amazon and Google Adsense.

What this means for you:

- I do not and will not recommend a product just because I'm an affiliate.
- I became an affiliate to earn some revenue towards the costs of running and maintaining this website. Where I have direct control over which ads are served on this website I offer only products that are directly related to the topic of this website and products that a reader/subscriber would have a genuine interest in or need of.
- I do not recommend products just for the sake of making money.
- I recommend products because I think they are valuable to my readers.
- I do not let the compensation I receive influence the content, topics, posts, or opinions expressed on this website.
- I respect and value my readers too much to write anything other than my own genuine and objective opinions and advice.

Just like this website, my Disclosure Policy is a work in progress. As the revenue streams evolve, so will this page.

## License

This theme is free and open source software, distributed under the MIT License. So feel free to use this Jekyll theme on your site without linking back to me or including a disclaimer.

---

Contact: vitess@googlegroups.com  **·**  
Announcements  **·**  
© 2017 Vitess powered by Google Inc
